# CMR in inflammatory vasculitis

**DOI:** 10.1186/1532-429X-14-82

**Published:** 2012-11-30

**Authors:** Subha V Raman, Ashish Aneja, Wael N Jarjour

**Affiliations:** 1 The Ohio State University, 473 W. 12th Ave, Suite 200, Columbus, OH, 43210, USA; 2 Division of Cardiovascular Medicine, The Ohio State University, 473 W. 12th Ave, Suite 200, Columbus, OH, 43210, USA; 3 Division of Rheumatology, The Ohio State University, 480 Medical Center Drive, S2056 DMRC, Columbus, Oh, 43210, USA

**Keywords:** Vasculitis, Magnetic resonance, Angiography, Inflammation, Imaging

## Abstract

Vasculitis, the inflammation of blood vessels, can produce devastating complications such as blindness, renal failure, aortic rupture and heart failure through a variety of end-organ effects. Noninvasive imaging with cardiovascular magnetic resonance (CMR) has contributed to improved and earlier diagnosis. CMR may also be used in serial evaluation of such patients as a marker of treatment response and as an indicator of subsequent complications. Unique strengths of CMR favoring its use in such conditions are its abilities to noninvasively visualize both lumen and vessel wall with high resolution. This case-based review focuses on the large- and medium-vessel vasculitides where MR angiography has the greatest utility. Because of increasing recognition of cardiac involvement in small-vessel vasculitides, this review also presents evidence supporting greater consideration of CMR to detect and quantify myocardial microvascular disease. CMR’s complementary role amidst traditional clinical, serological and other diagnostic techniques in personalized care for patients with vasculitis is emphasized. Specifically, the CMR laboratory can address questions related to extent and severity of vascular involvement. As ongoing basic and translational studies better elucidate poorly-defined underlying molecular mechanisms, this review concludes with a discussion of potential directions for the development of more targeted imaging approaches.

## Introduction

Vasculitis, the inflammation of blood vessels, is rare [1,2], yet those affected may suffer high rates of morbidity and mortality. A significant contributor to poor outcomes is delay in diagnosis. While advances in serologic testing have afforded somewhat earlier diagnosis, there remains a need for better techniques to detect vasculitis and improve outcomes. Advances in non-invasive imaging are poised to fill this gap, particularly in vasculitis involving large- and medium-sized arteries where these studies can play a significant role in i) establishing the correct diagnosis, ii) determining disease extent and activity and iii) measuring treatment response [3].

Recognition that not only vascular but also cardiac sequelae occur in certain vasculitides makes cardiovascular magnetic resonance (CMR) appealing amidst the various imaging options available [4]. Some clinicians may not consider CMR due to concerns surrounding the use of gadolinium-based contrast in vasculitis patients who frequently present with significant renal dysfunction. The availability of excellent non-contrast MR angiography techniques can allay such concerns, allowing CMR in high-risk patients who may have the most to benefit from timely use of the technology.

The paucity of well-defined pathogenic mechanisms for many of these disorders renders in some respect inadequate each of the many vasculitis classification schemes. In this review, we use the common approach that focuses on the sizes of the involved blood vessel that can guide CMR protocols and afford differential diagnosis of imaging findings. This approach, however, requires an appreciation that while specific types of vasculitis classically affect certain-sized blood vessels, there remains a spectrum of involvement across the various levels of the vasculature. What follows is a brief summary of the techniques most relevant to the CMR vasculitis examination followed by case-based illustrations of the major primary inflammatory vasculitides.

While this review focuses on the primary vasculitides, secondary vasculitis and mimics of vasculitis warrant consideration when evaluating a patient with suspected vasculitis [5]. Secondary vasculitis may occur in the setting of infection, malignancy, connective tissue disease [6], drugs and environmental exposure. Another consideration with vasculitis of medium and small vessels is primary central nervous system (CNS) angiitis or involvement of the CNS with a systemic vasculitis. This, too, is beyond the scope of this review but can be pursued in greater depth in several recent publications of MR’s role in the diagnosis and serial assessment of cerebral vasculitis [7,8].

### CMR techniques in the vasculitis examination

**Figure 1:**
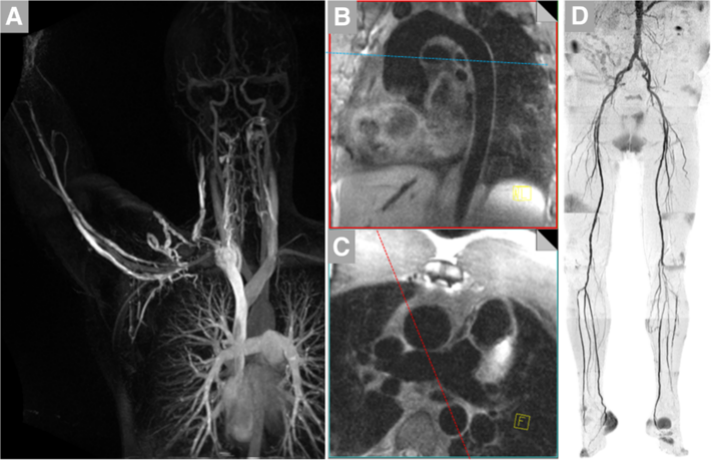
**CMR provides a variety of techniques for noninvasive, three-dimensional imaging of the entire vascular tree that may be useful in patients with known or suspected vasculitis such as contrast-enhanced bright blood angiography.** (**A**), noncontrast dark blood angiography (**B** and **C**, with lines indicating planes of reformatting of data from the 3D acquisition) and noncontrast bright blood angiography (**D**, shown in inverted grayscale).

**Figure 2:**
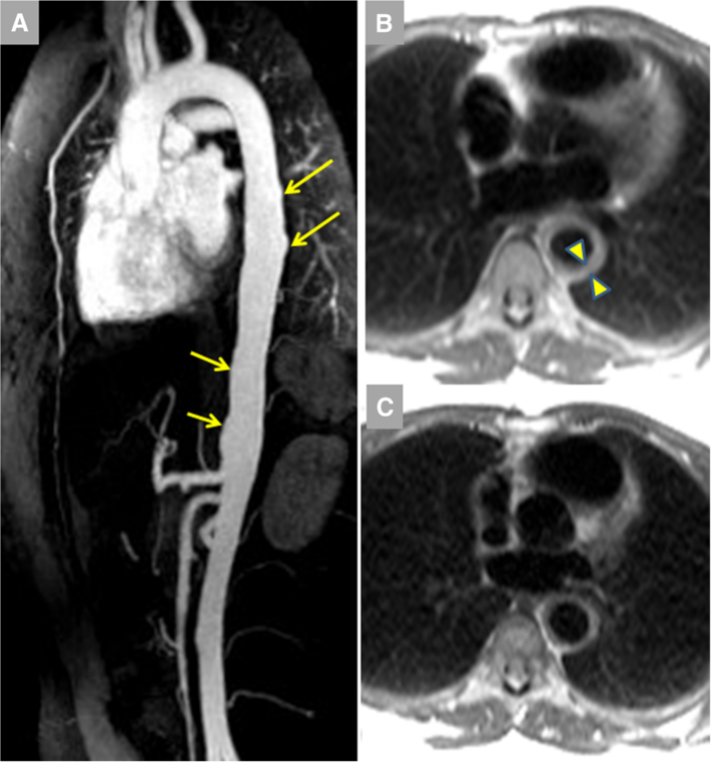
**Vasculitis was identified in a 28 year-old female with unrelenting back pain initially referred for MR examination to rule out aortic dissection; additional history revealed recent unintentional 5 kg weight loss.A**. Contrast-enhanced magnetic resonance angiography (CE-MRA) showed diffuse luminal irregularities (arrows). **B**. Pre-contrast dark blood imaging indicated marked aortic wall thickening to 9–10 mm (arrowheads). There was marked elevation of inflammatory markers including erythrocyte sedimentation rate (ESR, 94 mm/hr) and c-reactive protein (7.3 mg/L) levels. Symptoms markedly improved with prednisone, with reduced ESR (12 mm/hr) and aortic wall thickness at 12-month follow-up.

The evaluation of vasculitis by CMR may include angiography, vessel wall imaging and occasionally myocardial assessment (Figure 1, Table 1). The typical patient presenting with a vasculitis query to the CMR laboratory is one whose clinical, serological and even histopathological evaluations may have already led to diagnosis of a particular condition. For instance, a patient who initially presented with classic signs and symptoms of giant cell arteritis (GCA), underwent definitive temporal artery biopsy and received prednisone therapy, may subsequently be referred for CMR to define the extent of aortic involvement. Less commonly, the CMR laboratory is the first to propose vasculitis as a mechanism underlying the observed findings (Figure 2).

**Table 1:** Components of the CMR Examination of Vasculitis

**Technique**	**Comments**
PRECONTRAST	
Dark blood stacks typically in axial, coronal and sagittal planes e.g. HASTE	Provides vessel wall imaging as well as complementary information to CE-MRA regarding lumen
Noncontrast bright blood stack(s) e.g. SSFP	
	CONTRAST	
3D contrast-enhanced magnetic resonance angiography e.g. spoiled gradient echo	Appropriate vasculature should be covered depending on clinical questions and known or suspected diagnosis (see Table 2)	
	POSTCONTRAST	
T1-weighted vessel wall imaging e.g. VIBE or FAME	Additional vessel wall imaging, particularly useful to delineate thickening and thrombus	
	CARDIAC ACQUISITIONS	
Multiplane cine imaging e.g. SSFP Aortic valve velocity-encoded cine Myocardial imaging: T2 precontrast, T1W early post contrast, late post-gadolinium imaging	May be appropriate when aortic root disease involves the aortic valve or when myocardial inflammation is suspected, particularly in small-vessel vasculitides	

Techniques for angiography include both contrast-enhanced MR angiography (CE-MRA) as well as non-contrast approaches, both recently summarized in this journal by Hartung *et al*[9]. MRA’s sensitivity is typically at or near 100% while specificity tends to be lower, underscoring the potential to overestimate degree of stenosis particularly in branch vessels of the aorta. The workhorse technique for demonstration of luminal stenosis remains CE-MRA, with improved spatial resolution, better vessel-to-background contrast and reduced volume requirement for exogenous contrast material with higher field strength. These advantages offset limitations imposed by radiofrequency field inhomogeneity that increases with field strength. Technical developments in coil hardware and parallel acquisition techniques make higher field scanners appealing platforms for CE-MRA.

Non-contrast approaches for angiography abound, starting with traditional time-of-flight (TOF) angiography. While infrequently relied upon for stenosis assessment in extracranial arteries, TOF angiography does provide complementary information to CE-MRA. Rapid, lower-resolution 3D TOF imaging prescribed over a large volume helps identify sites of disease for higher-resolution 2D dark blood scans for vessel wall imaging. Our laboratory and many others have found 3D navigator SSFP imaging to be tremendously useful for non-contrast bright blood MRA of the thoracic aorta [10], particularly when CE-MRA may not be feasible due to contraindications to gadolinium-based contrast administration. Similarly, 3D non-contrast dark blood MRA may be useful in certain settings, particularly when higher heart rate, greater field strength or other approaches help shorten what are typically longer scan times [11]. Dark blood imaging is also preferable when susceptibility artifact obscures interpretation of bright blood images. Another SSFP-based non-contrast approach that is particularly appealing for lower extremity imaging is non-enhanced quiescent-interval single-shot MR angiography (Figure 1) [12]. While initially developed for atherosclerosis imaging of the peripheral arteries, it warrants consideration in vasculitides where peripheral vascular involvement is not uncommon. Some of the more commonly used elements of the CMR vasculitis protocol are listed in Table 1. Table 2 suggests which segments of the arterial tree should be imaged in the most common primary vasculitides. Table 3 summarizes studies comparing magnetic resonance to other modalities commonly used to evaluate the inflammatory vasculitidies.

**Table 2:** Typical arterial segments involved in the major primary vasculitides

	**Thoracic Aorta**	**Abdominal Aorta**	**Pulmonary Arteries**	**Carotid Arteries**	**Upper Extremities**	**Mesenteric arteries**	**Renal arteries**	**Lower Extremities**	**Coronary Arteries**
**Giant cell arteritis**	X	X		X	X				
**Takayasu arteritis**	X	X	X	X	X				
**Polyarteritis nodosa**						X	X		
**Kawasaki disease**									X
**Behçet disease**			X					X	

The most common conditions prompting referral for MR examination are shown with X indicating typically-involved segments of the extracranial arterial tree. This scope warrants consideration when prescribing the imaging protocol. Note that atypical manifestations have been reported in virtually all vessel territories for these disorders.

**Table 3:** MRI in inflammatory vasculitidies

**Author (Reference #)**	**Year published**	**Design**	**Comparison Groups**	**N**	**Role of CMR**
**Giant Cell Arteritis**					
Kornigkam-Santos [21]	2011	Retrospective	MRA in patients vs. retrospective normal controls	28	MRA detected GCA in 67% with good inter-observer agreement
Bley [25]	2005	Prospective	MRA vs. Biopsy	20	16/17 GCA + by biopsy were MRA +, all 3 GCA – were MRA -
Bley [26]	2005	Unclear	MRA in diagnosed GCA patients	21	MRA demonstrated vascular involvement in all previously diagnosed 9 patients
Walter [27]	2005	Unclear	PET in GCA	30 patients and 31 controls	PET had a sensitivity of 73.3% and specificity of 83.9%
Meller [28]	2005	Unclear	PET vs. MRA	15 FUO patients	MRA and PET had comparable sensitivity and specificity for detecting inflammation. Identical vascular territories were identified in the majority but disparate territories in a large minority
Cyran [29]	2011	Prospective	PET vs. MRI	17	Both Dynamic Contrast Enhanced MRI and PET had identical sensitivity and specificity (86 and 90% respectively) in assessing carotid and vertebral inflammation
Both [31]	2008	Prospective	PET vs. MRI	25	MRI and PET found unreliable for assessing large-vessel inflammation in GCA patients on pre-existing immunosuppressive therapy
**Takayasu’s Arteritis**					
Li [37]	2011	Retrospective	Whole body MRI	42	Wall thickness and post-contrast intensity by MRI higher in active group than remissive group (6.12 vs. 4.31 mm and 1.56 vs. 1.17)
Desai [38]	2005	Retrospective case series	MRA-inversion recovery prepared gradient-echo MR pulse sequence	7	All patients had increased wall thickness and 5 had enhancement with contrast (4 had clinically active disease)
Choe [39]	2000	Prospective	MRI	26 patients and 16 controls	MR imaging was concordant with clinical findings in 23 patients (88.5%), with laboratory findings in most patients (ESR in 92.3% [24/26] and CRP in 84.6% [22/26])
Jiang [40]	2012	Prospective	MRA	26 patients-16 classified as active and 10 as inactive	Active group had more stenosis in left SCA than the inactive group (14/16, 87.5% vs. 2/10, 20%; p<0.01) greater vessel wall thickness in left CCA (11/16, 68.75% vs. 1/10, 10%; p<0.01) and left SCA (9/16, 56.25% vs. 0/10, 0%; p<0.01)
Tso [41]	2002	Retrospective	MRA	24	MRA revealed vessel wall edema in 94% (17 of 18), 81% (13 of 16), and 56% (24 of 43) of studies during periods of unequivocally active disease, uncertain disease activity, and apparent clinical remission, respectively. ESR and CRP did not correlate with clinical assessment or MR evidence of vascular edema
Yamada [34]	2000	Prospective	MRA vs. CA	30	Takayasu arteritis was diagnosed in 20 patients - MRA accurately revealed 323 (98%) of 330 arteries, but 7 (2%) stenotic lesions were overestimated as occluded. The sensitivity and specificity of MRA for diagnosis of Takayasu arteritis were 100%. PA lesions were demonstrated in 10 (50%) of the 20 patients.
Garg [35]	2011	Prospective	MRA vs. DSA	22	Diagnosis confirmed by MRA in all patients. MRA with sensitivity, specificity, PPV, NPV and DA for detection of a >50% lesion was 98.33%, 97.25%, 92.18%, 99.43% and 97.52% respectively.
**Kawasaki Disease**					
Greil [54]	2007	Prospective	MRA vs. CA	6	Complete agreement between MRA and CA in detection of coronary aneurysms (n=15).Excellent agreement for aneurysm diameter, length, and distance from the ostium.
Tacke [55]	2011	Prospective	MRA vs. Echocardiography	63	MRA detected coronary aneurysms in 15 patients, whereas echo detected aneurysms in 11.
Greil [56]	2002	Prospective	MRA vs. CA	6	Excellent agreement for assessment of coronary aneurysm maximal diameter and length
Mavrogeni [57]	2004	Prospective	MRA vs. CA	13	6 patients had coronary aneurysms and 7 had ectasia. MRA and CA agreed completely for the diagnosis of aneurysms
Suzuki [58]	2006	Retrospective	MRA vs. CA	106	MRA agreed well with CA for detecting aneurysms and stenoses
Arnold [59]	2007	Prospective	MRA vs. Multidetector CT vs. CA	16	100% agreement between MDCT and CA in the detection of aneurysms and stenoses. MRI and CA had 93% agreement for the detection of aneurysms. MRI missed one stenosis.
Mavrogeni [61]	2011	Unclear	MRA in Kawasaki disease patients in convalescence	13	MRA revealed high prevalence of coronary ectasia and myocarditis in 46% (n=13) of convalescing Kawasaki disease patients

Abbreviations. MRA: magnetic resonance angiography, PET: positron emission tomography, MRI: Magnetic resonance imaging, ESR: erythrocyte sedimentation rate, CRP: C-reactive protein, SCA: subclavian artery, CCA: common carotid artery, CA: conventional angiography, PPV: positive predictive value, NPV: negative predictive value, DA: diagnostic accuracy.

### Large vessel vasculitis

#### Giant cell arteritis (GCA)

GCA is a granulomatous, large-vessel vasculitis with a predilection for individuals aged ≥70 years. Up to 60% of patients with GCA have findings consistent with polymyalgia rheumatica such as proximal muscle morning stiffness and aching [13]. The overall incidence is highest in individuals of Scandinavian descent, where it occurs in 18 to 20 per 100,000 individuals [14]. GCA can be diagnosed as present if 3 of the following 5 classical findings are observed: i) age ≥50 years; ii) new-onset or new type of localized pain in the head; iii) temporal artery tenderness; iv) erythrocyte sedimentation rate ≥50 mm/h; and v) arterial biopsy specimen showing vasculitis [15]. However, appreciating that non-classic symptoms may be the initial manifestation of GCA, high clinical suspicion and rapid institution of, for instance, steroid therapy is needed to avoid dreaded complications such as vision loss. Examination of the retina with fluorescein angiography holds particular value in diagnosis [16].

Classically, GCA affects the extracranial branches of the carotid artery, notably the temporal artery. Risk of developing thoracic and abdominal aortic aneurysms is 17 and 2 times higher, respectively, in patients with GCA compared to age-matched individuals without GCA [17]. Aortic involvement in GCA carries a very high risk of rupture and death, with an average survival of 1 year in GCA patients with thoracic aortic dissection [18]. This considerable increase in mortality due to aortic involvement mandates a low threshold for aortic imaging in this disease [19,20]. Subclavian artery and axillary artery involvement are not uncommon in GCA, and MR performs well in delineating such [21]. GCA affected lower extremities alone in 17% of patients in a recent series [22]. Recognition of long stenotic segments and vessel wall thickening with MR can prompt specific treatment (e.g., prednisone) in such GCA patients who may present solely with claudication. Follow-up MR may identify reduced vessel wall thickening indicating a favorable response to therapy [4].

The sensitivity and specificity of Doppler ultrasonography (US) compared to the clinical diagnosis of temporal arteritis in a meta-analysis of 23 studies was 69 and 82% respectively [23]. Additionally, since US assessment was confined to detection of the “halo” sign in the temporal artery, it cannot reliably assess the thoracic aorta, whose involvement can lead to the serious and fatal complications as discussed above. Nonetheless, European League Against Rheumatism (EULAR) guidelines assign US a class Ia recommendation for the diagnosis of GCA vs. IIa for MRA [24]. Alternatively, 3T MRI of the cranial arteries has a high sensitivity and specificity in detecting vessel wall inflammation when compared with temporal artery biopsy (89-94%, and 92-100% respectively), albeit in small studies [25,26]. Positron emission tomography (PET) may have a higher sensitivity than MRI in detecting GCA according to some retrospective studies [27,28]. However, PET is limited in its ability to demonstrate temporal artery inflammation because of inadequate spatial resolution and inability to distinguish luminal changes in close proximity to glucose-avid brain tissue. In addition, it involves considerable exposure to ionizing radiation. Recently, high resolution dynamic contrast-enhanced (DCE) MRI performed well in detecting vessel inflammation in patients with suspected arteritis [29]. The abnormal extraction fraction computed from first-pass MR imaging, implying increased endothelial leakiness, correlated well with FDG-uptake in arterial walls by PET-CT.

Current data suggest no clear role for either CMR or PET in GCA patient follow up. PET does detect decline in activity with treatment, but in most cases the uptake does not disappear. This is thought to arise from chronic changes associated with vessel wall remodeling. In one GCA study including patients with a complicated course, PET findings correlated poorly with clinical and laboratory criteria of disease activity and MRI findings. In the same study, PET could not predict disease relapse [30]. Of note, MRI with dark blood imaging may demonstrate wall thickening and edema that persists despite clinical and laboratory improvements [31].

#### Takayasu arteritis (TA)

TA shares many of the vascular findings of GCA but occurs in considerably younger patients, classically women more often than men under the age of 50 years. Diagnosis requires angiographic abnormalities [32] plus one of the following four findings: i) decreased peripheral artery pulse(s) and/or claudication of extremities; ii) blood pressure difference >10 mm Hg between extremities; iii) bruits over aorta and/or its major branches; or iv) hypertension. The mandate for angiographic evidence of disease makes MR an important component of the TA evaluation, especially in children where invasive angiography and CT hold considerably less appeal.

**Figure 3:**
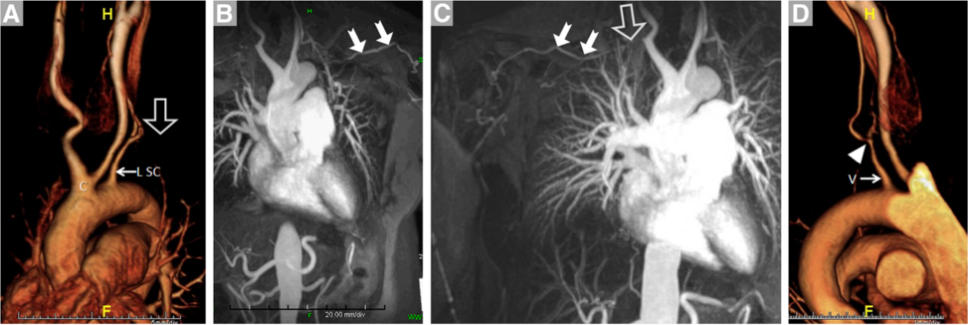
**A 42 year-old female presented with bilateral arm fatigue, worse with lifting above the head.** Physical examination showing absent radial pulses, and serum inflammatory markers including erythrocyte sedimentation rate and c-reactive protein levels were elevated. Anemia was also present (hematocrit 30%). With a presumptive diagnosis of Takayasu arteritis, treatment with prednisone was initiated and CE-MRA was requested. **A**. Volume rendering shows patency of the common brachiocephalic trunk (C); the proximal portion of the left subclavian artery (L SC, arrow) is patent while distally it is occluded (open arrow). **B**. Maximum intensity projection (MIP) shows reconstitution of the distal L SC (arrows) via collaterals. **C**. Similarly, a MIP image shows that the right subclavian artery is occluded (open arrow) and fills distally (filled arrows) via collaterals. **D**. Volume rendering demonstrates high-grade stenosis (arrow) of the left vertebral artery (V).

TA, previously termed “pulseless disease” because bilateral subclavian artery occlusion eliminates the radial pulse (Figure 3), can also affect the aorta, pulmonary arteries and renal arteries; some data suggest a predilection for bilateral vessel involvement [33]. In a direct comparison of MRI with invasive angiography, Yamada *et al.* demonstrated accurate evaluation of 98% vascular territories and also demonstrated pulmonary artery involvement in 50% of patients, identical to the distribution delineated by conventional angiography [34]. Similarly, Garg *et al.* have also shown a high correlation between MRA and digital subtraction angiography in detecting stenosis and aneurysm formation [35].

**Figure 4:**
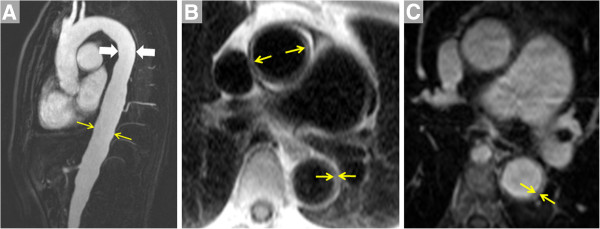
**Images of the thoracic aorta in a patient with Takayasu arteritis are shown.A**. CE-MRA in the sagittal plane demonstrates diffuse, mild dilatation of the descending aorta that measured 33 mm at the level indicated by arrows vs. 25 mm more proximally (arrowheads). Vessel wall thickening can be appreciated using techniques such as non-contrast inversion recovery dark blood imaging (**B**, showing thickening of 4–5 mm of the thoracic aorta wall, arrows). Additional post-contrast T1-weighted imaging such as the volumetric interpolated breathhold technique (**C**, same location as **B**) further confirm vessel wall thickening in this patient.

**Figure 5:**
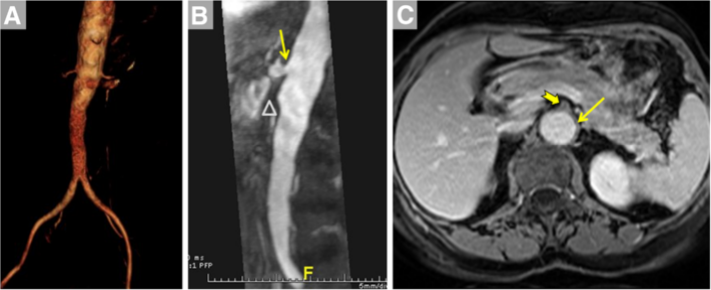
**Images of the abdominal aorta in a patient with Takayasu arteritis are shown.A**. Volume rendering viewed in the antero-posterior direction of a CE-MRA acquired using a coronal slab demonstrates marked irregularity of the infraceliac abdominal aorta. **B**. Maximum intensity projection of a CE-MRA obtained using a sagittal slab at a subsequent visit shows ostial disease of the celiac trunk (arrow) and absence of the superior mesenteric artery (SMA, open triangle). **C**. Post-contrast volumetric interpolated breathhold T1-weighted image shows thickening of the wall of the abdominal aorta (arrow) and obliteration of the SMA ostium (notched arrow).

Using MR’s ability to assess vessel wall thickening and disease activity, Andrews *et al.* underscored the utility of MR over traditional x-ray angiography in facilitating early diagnosis and guiding treatment [36]. Given the extent of potential arterial involvement and the requirement for accurate diagnosis (Figures 3, 4, 5), complete aortic imaging with visualization of all major branches should be performed in every TA patient [13]. Li and colleagues demonstrated the diagnostic utility in patients with confirmed TA of whole-body MRA combined with dark blood axial plane vessel wall imaging from neck to pelvis [37]. A correlation with disease activity (clinical and laboratory) with inflammation on MRI was demonstrated by Desai *et al.*, Choe *et al.*, and Jiang *et al.* using primarily pre-contrast T1W and T2W dark blood imaging but also post-gadolinium T1W imaging [38-40], but not replicated by Tso *et al.*, who with T2W-STIR demonstrated evidence of inflammation by MRI when the disease was clinically quiescent [41]. With histological evidence of ongoing inflammation in Takayasu patients when the disease is clinically quiescent [42], imaging-based detection of vessel wall inflammation warrants further evaluation in clinical trials that examine the effect of treating subclinical disease on outcomes.

#### Idiopathic aortitis

**Figure 6:**
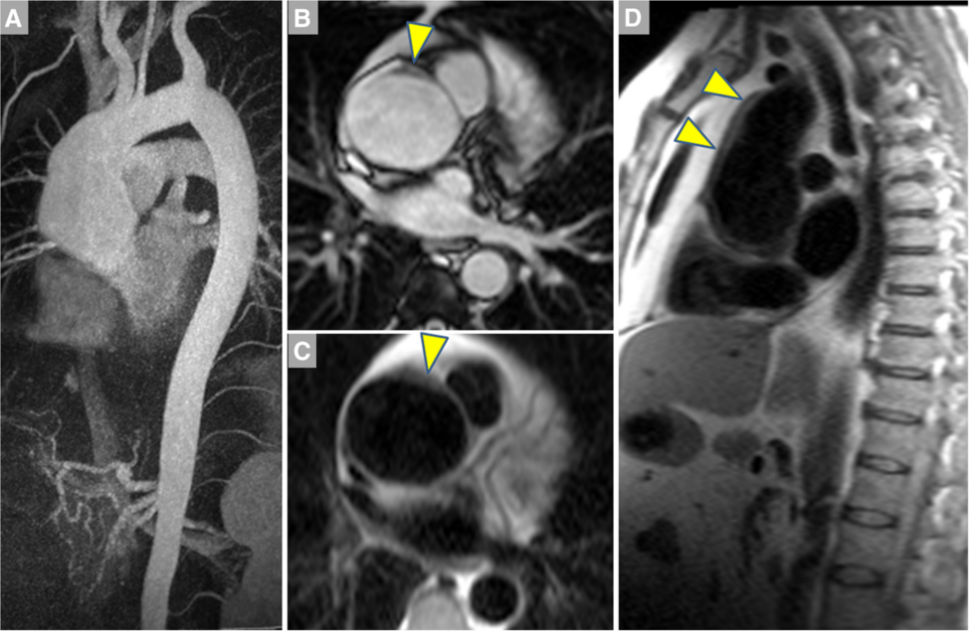
**A 58 year-old male with fatigue and palpitations underwent transthoracic echocardiography that indicated dilatation of the aortic root.** CMR was ordered to assess the aorta. **A**. MIP of the CE-MRA shows marked dilatation of the ascending aorta, which measured up to 6 cm in diameter compared to the 2.5 cm arch. **B**. Single heartbeat true FISP bright blood image shows thickening of the aortic wall (arrowhead), also evident on HASTE dark blood imaging in the axial (**C**) and sagittal planes (**D**).

**Figure 7:**
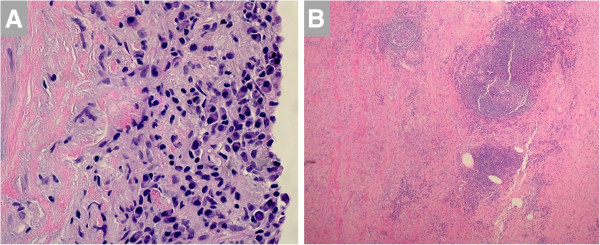
**A. Histopathological examination of the aortic wall in a patient with idiopathic aortitis demonstrates an abundance of plasma cells in the intima.B**. Low power light microscopy demonstrates inflammatory cells in media (right side) and adventitia (left side). Warthin-Starry stain was negative for spirochetes. Images courtesy of Drs. Mark Brownell and Anne Albers.

**Figure 8:**
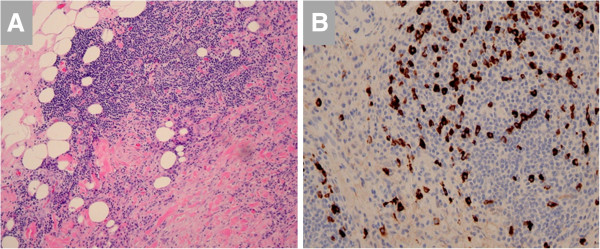
**A. Inflammatory infiltrate in a patient with idiopathic aortitis is seen to extend from the adventitia into the mediastinal fat.B**. Immunohistochemistry demonstrates IgG4-positive plasma cells. Images courtesy of Drs. Mark Brownell and Anne Albers.

This inflammatory, noninfectious aortitis has been described both as an isolated aortic disease at one end a spectrum that includes GCA-TA at the other end [43]. However, unlike GCA and TA, idiopathic aortitis infrequently presents with constitutional symptoms but more likely manifests as aortic aneurysm in the setting of asthma or eczema [44]. With recognition that tissue-infiltrating IgG4-positive plasma cells represent the *sine qua non* of this disease [45], it has been more appropriately included in the spectrum of IgG4-mediated disorders [46]. Typical manifestations include aneurysms of the ascending thoracic or abdominal aorta (Figure 6), and two large studies reported a prevalence of 4 to 6% when examining surgical aortic specimens [47,48]. When histology is available, the diagnosis can be made by demonstration of an abundance of plasma cells spanning the intima, media and adventitia and by their positive staining for IgG4 (Figures 7, 8). An association with retroperitoneal fibrosis has also been described that may lead to ureteral obstruction. CMR in a patient with idiopathic aortitis should span the thorax and abdomen, with attention paid not only to aortic dimensions and wall morphology but also other potentially affected abdominal organs [46].

### Medium vessel vasculitis

#### Kawasaki disease (KD)

This leading cause of acquired heart disease in children begins with fever persisting for at least four days. Additionally, diagnosis requires 4 of the following 5 features: i) desquamative rash of the extremities or perineal area; ii) polymorphous exanthema; iii) bilateral conjunctival injection; iv) injection of oral and pharyngeal mucosa; and v) cervical lymphadenopathy [32,49]. Involvement of the coronary arteries produces much of KD’s mortality and morbidity via myocardial infarction and heart failure. Coronary artery aneurysms develop in up to 25% of untreated children [50]; intravenous immunoglobulin therapy reduces this incidence to 3 or 4% [51].

**Figure 9:**
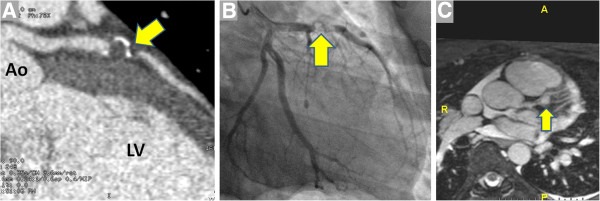
**An 11 year-old boy presented to a pediatric hospital with chest and jaw pain while playing one year after a prolonged febrile illness.** Initially, CT angiography was performed (**A**, image courtesy of Dr. Christopher Learn) that showed thrombus in a calcified aneurysm of the left anterior descending coronary artery (LAD, arrow). In the setting of elevation of the serum troponin and possible need for coronary intervention, the patient was transferred to a nearby adult hospital. Invasive angiography (**B**) showed thrombus nearly occluding LAD that was treated with angioplasty and stent placement. **C**. Coronary MRA performed in another patient with KD using a navigator-triggered slab prescribed perpendicular to the aortic root demonstrates a 9 mm proximal LAD aneurysm (arrow). LV = left ventricle Ao = aorta.

**Figure 10:**
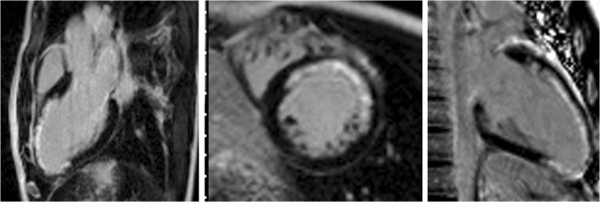
LGE-CMR in three-chamber (left), mid short-axis (center) and vertical long axis (right) planes show LAD-territory infarct scar in a boy with Kawasaki disease.

As cardiovascular death has been reported up to 18 years after the initial illness [52], both pediatric and adult cardiovascular specialists should be attuned to the essential elements of follow-up evaluation. The initial evaluation of the child with acute KD includes echocardiography for coronary artery imaging, which is particularly feasible at high volume centers and in smaller children who receive sedation [53]. Because the presence and extent of coronary artery involvement dictate follow-up treatment and testing [49], poor visualization by echo should prompt consideration of CMR for coronary artery imaging in KD [54]. CMR’s lack of ionizing radiation and noninvasive nature make it preferable in children and young adults with KD over modalities such as computed tomography and invasive x-ray angiography (Figures 9, 10). Tacke *et al.* recently demonstrated the utility of a more comprehensive assessment afforded by CMR that included rest and stress perfusion imaging, cine imaging for wall motion abnormality and late post-gadolinium imaging for infarct scar [55]. Greil *et al.* first demonstrated equivalence of MRI with conventional angiography in detecting coronary aneurysms, stenosis and complete occlusions using a free-breathing, T2-prepared, 3D bright-blood sequence with navigator gating and tracking [56]. Subsequently, Mavrogeni *et al.* confirmed complete agreement between conventional angiography and this MRA technique in demonstrating coronary ectasia and aneurysms [57]. Using bright-blood and dark-blood imaging, Suzuki *et al.* replicated the equivalence of MRI with conventional angiography and superiority over echocardiography in detecting aneurysms and stenosis in patients ranging from 4 months to 37 years [58].

Arnold *et al.* compared conventional angiography, multi-detector CT (MDCT) and MRI in 16 patients and showed 100% concordance between conventional angiography and MDCT, and 93% concordance between MRI and coronary angiography for aneurysm detection [59]. MRI missed one stenotic lesion, while providing additional information regarding myocardial inflammation and injury. Kan *et al.* recently summarized the safety and accuracy of contemporary CT with ultra-low radiation exposure for pediatric patients [60], including 5 with suspected coronary involvement in KD with nondiagnostic CMR examination. More recently, using free-breathing, T2-prepared, 3D-SSFP, whole heart approach with navigator gating and tracking, Greil *et al.* demonstrated complete and excellent agreement between MRA and coronary angiography [54].

Mavrogeni *et al.* reported comprehensive CMR findings using both coronary and myocardial evaluation in 13 KD patients thought to be in convalescence (stage II and III, 20–25 days after disease onset) [61]. Of the six patients with demonstrable myocardial inflammation by T2W STIR (which had resolved in all 6 by repeat CMR 3 months later), only three had evident abnormality by LGE yet all had lower LV ejection fraction by cine imaging. Additional 3D ECG-gated steady state free precession navigator-gated non-contrast coronary MRA identified coronary aneurysms and intracoronary thrombus, some of which were missed by echocardiography. In joint guidelines issued by the American Academy of Pediatrics and the American Heart Association in 2004, echocardiography, MRI, and conventional coronary angiography were all accorded level of evidence C for detection of coronary abnormalities due to lack of prospective data [62].

#### Polyarteritis nodosa (PAN)

Whereas KD is considered an acute medium vessel vasculitis, PAN is typically a subacute disorder affecting adults more than children. While all the vasculitides are relatively rare, higher incidences of PAN have been reported in Alaskan and Kuwaiti natives [1]. PAN classically affects visceral arteries such as renal, hepatic and mesenteric arteries. Presenting findings often include hypertension, fever, musculoskeletal symptoms, abdominal angina and neuropathy, occasionally with hepatitis B antigen or DNA in the serum [63].

**Figure 11:**
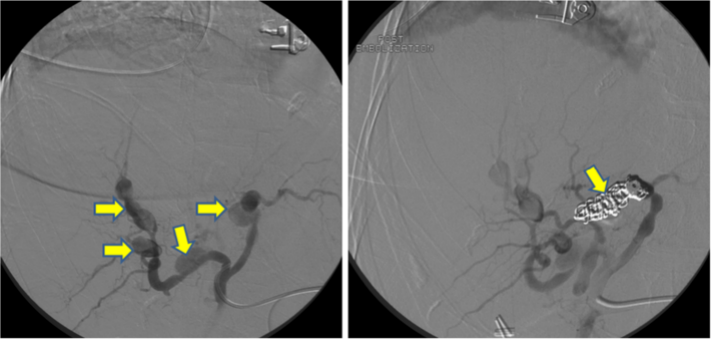
**Traditional x-ray angiography may still be employed in most centers to diagnose polyarteritis nodosa involving the visceral arteries, such as the focal, segmental aneurysms (left panel, up to 4.5 cm in diameter) of the hepatic artery seen in this 77 year-old woman with acute abdominal pain, syncope and bleeding within the liver parenchyma by CT.** The angiographic appearance was consistent with PAN. Coil embolization (right panel) was performed with resolution of bleeding. Images courtesy Dr. Leslie Cooper.

While invasive x-ray angiography may traditionally be used to demonstrate renal artery involvement in PAN, especially when therapy via coil embolization is being considered (Figure 11), CMR may uncover both coronary involvement and its sequelae. Mavrogeni and colleagues identified coronary artery aneurysms in 4 of 16 PAN patients using 3D whole heart navigator MRA [64], and resultant myocardial injury was demonstrated in a patient with PAN by Kobayashi *et al.* using T2-weighted and LGE imaging of the myocardium [65]. In a longitudinal study of patients with immune-mediated disease comparing incident coronary heart disease (CHD) hospitalization to the entire population of Sweden, Zöller *et al.* found in 248 patients with PAN a standardized incidence ratio for CHD of 3.81 (95% CI 2.72–5.19) in the first year after PAN hospitalization [66]. Prospective studies are needed to better define the utility of CMR in earlier detection and targeted intervention to prevent CHD complications in PAN.

#### Behçet disease (BD)

**Figure 12:**
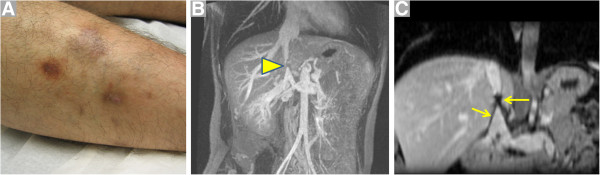
**A 25 year-old Middle Eastern male with a 6 year history of recurrent deep venous thrombi despite therapeutic anticoagulation presented to a vascular medicine specialist.** Physical examination demonstrated painful erythematous lesions over the lateral aspect of the calf (**A**, Courtesy Dr. Steven Dean) and oral ulcerations. **B**. Subsequent contrast-enhanced magnetic resonance venogram showed occlusion of a previously placed filter in the inferior vena cava (arrowhead). Extensive venous collaterals are evident. **C**. Coronal plane post-contrast volumetric interpolated breathhold T1-weighted image shows signal void delineating the IVC filter (arrows).

This disorder is notorious for involving both arteries and veins across vessel sizes, and typically affects young males of Asian and Eastern Mediterranean descent. Presentation with recurrent venous thrombi in a young male with aphthous ulcers and/or uveitis should raise one’s suspicion of BD (Figure 12). Of note, vascular involvement may precede the more classical findings [67].

Pulmonary artery aneurysms contribute to excess mortality via rupture; aggressive treatment with steroids, immunosuppression and occasionally surgery has reduced mortality [68]. Recognizing such, vascular imaging of the patient with BD should at some point include the pulmonary arteries. A recent study identified cardiac complications in 6% of patients with this disease including pericarditis, right ventricular thrombus, myocardial infarction and endomyocardial fibrosis [69] – all of which can be readily demonstrated with a suitably-prescribed CMR examination.

### Small vessel vasculitis: Churg-Strauss syndrome (CSS)

**Figure 13:**
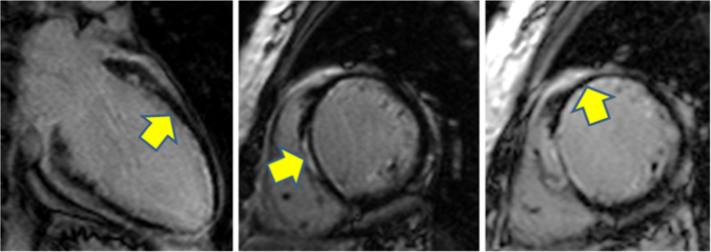
**A 37-year-old female with biopsy-proven Churg-Strauss-vasculitis was referred for CMR examination The left ventricle was slightly enlarged with mild systolic dysfunction: LV ejection fraction was 45%.** Late post-gadolinium myocardial enhancement images in various planes show septal intramural and anteroseptal and anterior subendocardial lesions. Images courtesy Drs. Ralf Waβmuth and Jeanette Schulz-Menger.

Patients with small vessel vasculitis have significant constitutional symptoms. Other more specific complaints are related to the particular bed that is affected. For example, palpable purpura is the hallmark dermatological manifestation of small vessel vasculitis, whereas alveolar hemorrhage may occur if the lung parenchyma is involved. Clinical clues to the diagnosis of CSS may include asthma, hypereosinophilia and fever, and half of these patients test positive for anti-neutrophilic cytoplasmic antibody(ANCA) [3]. CSS warrants particular attention in this review given that rates of cardiac involvement have been reported to be as high as 75 to 90%. Cardiovascular involvement in CSS consists of pericarditis, myocarditis, cardiomyopathies and intracavitary thrombi [70,71].In a series of 11 patients undergoing CMR, various forms of myocardial injury was detected in all patients. Mean LVEF was 45% with impairment of LV function in 6 patients, edema in 4, pericardial effusion in 7, and LGE-positivity in 9 including some with normal LV size and EF [72]. The subendocardial pattern of myocardial involvement in CSS can be readily detected by LGE-CMR but not echocardiography [73,74]. Existing data support the use of LGE and T2 myocardial imaging in defining the presence and extent of cardiac disease (Figure 13) [70,72]. Future studies using perfusion CMR may help elucidate to what extent microvascular disease contributes to myocardial involvement.

## Review and Conclusions

Su and colleagues, in one of the few studies of molecular imaging specifically targeting non-atherosclerotic vascular inflammation, showed feasibility of targeting the enzyme myeloperoxidase that is a marker of certain vasculitides [75]. Ongoing advances in understanding the molecular bases for these varied conditions may translate into novel diagnostic imaging approaches. Remarkably, diagnosis still heavily relies on direct tissue examination; development of MR agents that target IgG4 or ANCA could hopefully improve the diagnostic specificity of noninvasive imaging and reduce the need for histopathology.

In conclusion, diagnosis and serial assessment of patients with inflammatory vasculitis require clinical, laboratory and imaging assessments. The CMR laboratory in particular offers a spectrum of techniques for noninvasive angiography and vessel wall imaging, both with and without need for exogenous contrast administration. The utility of these techniques in patients with inflammatory vasculitis is realized only through 1) their timely use at initial presentation for accurate delineation of both presence and extent of disease and 2) an ongoing dialog among CMR specialists and the clinicians charged with initial diagnosis and long-term management. Such efforts coupled with continued advances in molecular understanding and treatment warrant further investigation given the considerable morbidity and mortality for patients with primary vasculitis.

## Competing interests

Dr. Raman receives research support from Siemens. Drs. Jarjour and Aneja have no competing interests to report.

## Authors’ contributions

SVR drafted the manuscript and figures. WNJ and AA contributed to manuscript preparation and revision. All authors read and approved the final manuscript.

## References

[B1] Scott DG Watts RA Systemic vasculitis: epidemiology, classification and environmental factors Ann Rheum Dis 2000 59 161 163 10.1136/ard.59.3.161 10700420 PMC1753099

[B2] Gonzalez-Gay MA Garcia-Porrua C Epidemiology of the vasculitides Rheum Dis Clin North Am 2001 27 729 749 10.1016/S0889-857X(05)70232-5 11723761

[B3] Jayne D The diagnosis of vasculitis Best Pract Res Clin Rheumatol 2009 23 445 453 10.1016/j.berh.2009.03.001 19508950

[B4] Pipitone N Versari A Salvarani C Role of imaging studies in the diagnosis and follow-up of large-vessel vasculitis: an update Rheumatology (Oxford) 2008 47 403 408 18292120 10.1093/rheumatology/kem379

[B5] Luqmani RA Pathare S Kwok-Fai TL How to diagnose and treat secondary forms of vasculitis Best Pract Res Clin Rheumatol 2005 19 321 336 10.1016/j.berh.2004.11.002 15857799

[B6] Mavrogeni S Vassilopoulos D Is there a place for cardiovascular magnetic resonance imaging in the evaluation of cardiovascular involvement in rheumatic diseases? Semin Arthritis Rheum 2011 41 488 496 10.1016/j.semarthrit.2011.04.004 21641017

[B7] Zuccoli G Pipitone N Haldipur A Brown RD Jr Hunder G Salvarani C Imaging findings in primary central nervous system vasculitis Clin Exp Rheumatol 2011 29 S104 S109 21586204

[B8] Berlit P Diagnosis and treatment of cerebral vasculitis Ther Adv Neurol Disord 2010 3 29 42 10.1177/1756285609347123 21180634 PMC3002614

[B9] Hartung MP Grist TM Francois CJ Magnetic resonance angiography: current status and future directions J Cardiovasc Magn Reson 2011 13 19 10.1186/1532-429X-13-19 21388544 PMC3060856

[B10] Krishnam MS Tomasian A Malik S Desphande V Laub G Ruehm SG Image quality and diagnostic accuracy of unenhanced SSFP MR angiography compared with conventional contrast-enhanced MR angiography for the assessment of thoracic aortic diseases Eur Radiol 2009 20 1311 1320 20013276 10.1007/s00330-009-1672-3 PMC2861759

[B11] 3rd Winner MW Raman SV Chung YC Simonetti OP Mihai G Cook SC Post-interventional three-dimensional dark blood MRI in the adult with congenital heart disease Int J Cardiol 2012 158 2 276 71 10.1016/j.ijcard.2011.01.050 21315462

[B12] Hodnett PA Koktzoglou I Davarpanah AH Scanlon TG Collins JD Sheehan JJ Dunkle EE Gupta N Carr JC Edelman RR Evaluation of peripheral arterial disease with nonenhanced quiescent-interval single-shot MR angiography Radiology 2011 260 282 293 10.1148/radiol.11101336 21502384 PMC3121010

[B13] Langford CA Vasculitis J Allergy Clin Immunol 2009 125 S216 225 19932919 10.1016/j.jaci.2009.07.002

[B14] Borchers AT Gershwin ME Giant cell arteritis: A review of classification, pathophysiology, geoepidemiology and treatment Autoimmun Rev 2012 11 A544 554 10.1016/j.autrev.2012.01.003 22285588

[B15] Hunder GG Bloch DA Michel BA Stevens MB Arend WP Calabrese LH Edworthy SM Fauci AS Leavitt RY Lie JT The American College of Rheumatology 1990 criteria for the classification of giant cell arteritis Arthritis Rheum 1990 33 1122 1128 2202311 10.1002/art.1780330810

[B16] Kale N Eggenberger E Diagnosis and management of giant cell arteritis: a review Curr Opin Ophthalmol 2010 21 417 422 10.1097/ICU.0b013e32833eae8b 20811283

[B17] Evans JM O'Fallon WM Hunder GG Increased incidence of aortic aneurysm and dissection in giant cell (temporal) arteritis A population-based study. Ann Intern Med 1995 122 502 507 10.7326/0003-4819-122-7-199504010-00004 7872584

[B18] Nuenninghoff DM Hunder GG Christianson TJ McClelland RL Matteson EL Mortality of large-artery complication (aortic aneurysm, aortic dissection, and/or large-artery stenosis) in patients with giant cell arteritis: a population-based study over 50 years Arthritis Rheum 2003 48 3532 3537 10.1002/art.11480 14674005

[B19] Bossert M Prati C Balblanc JC Lohse A Wendling D Aortic involvement in giant cell arteritis: current data Joint Bone Spine 2010 78 246 251 21030278 10.1016/j.jbspin.2010.09.013

[B20] Martinez-Valle F Solans-Laque R Bosch-Gil J Vilardell-Tarres M Aortic involvement in giant cell arteritis Autoimmun Rev 2010 9 521 524 10.1016/j.autrev.2010.02.014 20149900

[B21] Koenigkam-Santos M Sharma P Kalb B Oshinski JN Weyand CM Goronzy JJ Martin DR Magnetic resonance angiography in extracranial giant cell arteritis J Clin Rheumatol 2011 17 306 310 21869711 10.1097/RHU.0b013e31822acec6 PMC4271838

[B22] Assie C Janvresse A Plissonnier D Levesque H Marie I Long-term follow-up of upper and lower extremity vasculitis related to giant cell arteritis: a series of 36 patients Medicine (Baltimore) 2011 90 40 51 10.1097/MD.0b013e318206af16 21200185

[B23] Karassa FB Matsagas MI Schmidt WA Ioannidis JP Meta-analysis: test performance of ultrasonography for giant-cell arteritis Ann Intern Med 2005 142 359 369 15738455 10.7326/0003-4819-142-5-200503010-00011

[B24] Basu N Watts R Bajema I Baslund B Bley T Boers M Brogan P Calabrese L Cid MC Cohen-Tervaert JW EULAR points to consider in the development of classification and diagnostic criteria in systemic vasculitis Ann Rheum Dis 2010 69 10 1744 1750 10.1136/ard.2009.119032 20448283

[B25] Bley TA Wieben O Uhl M Thiel J Schmidt D Langer M High-resolution MRI in giant cell arteritis: imaging of the wall of the superficial temporal artery AJR Am J Roentgenol 2005 184 283 287 15615989 10.2214/ajr.184.1.01840283

[B26] Bley TA Weiben O Uhl M Vaith P Schmidt D Warnatz K Langer M Assessment of the cranial involvement pattern of giant cell arteritis with 3T magnetic resonance imaging Arthritis Rheum 2005 52 2470 2477 10.1002/art.21226 16052572

[B27] Walter MA Melzer RA Schindler C Muller-Brand J Tyndall A Nitzsche EU The value of [18F]FDG-PET in the diagnosis of large-vessel vasculitis and the assessment of activity and extent of disease Eur J Nucl Med Mol Imaging 2005 32 674 681 10.1007/s00259-004-1757-9 15747154

[B28] Meller J Strutz F Siefker U Scheel A Sahlmann CO Lehmann K Conrad M Vosshenrich R Early diagnosis and follow-up of aortitis with [(18)F]FDG PET and MRI Eur J Nucl Med Mol Imaging 2003 30 730 736 10.1007/s00259-003-1144-y 12677302

[B29] Cyran CC Sourbron S Bochmann K Habs M Pfefferkorn T Rominger A Raya JG Reiser MF Dichgans M Nikolaou K Quantification of supra-aortic arterial wall inflammation in patients with arteritis using high resolution dynamic contrast-enhanced magnetic resonance imaging: initial results in correlation to [18F]-FDG PET/CT Invest Radiol 2011 46 9 594 599 10.1097/RLI.0b013e31821c44ed 21577125

[B30] Blockmans D de Ceuninck L Vanderschueren S Knockaert D Mortelmans L Bobbaers H Repetitive 18F-fluorodeoxyglucose positron emission tomography in giant cell arteritis: a prospective study of 35 patients Arthritis Rheum 2006 55 131 137 10.1002/art.21699 16463425

[B31] Both M Ahmadi-Simab K Reuter M Dourvos O Fritzer E Ullrich S Gross WL Heller M Bahre M MRI and FDG-PET in the assessment of inflammatory aortic arch syndrome in complicated courses of giant cell arteritis Ann Rheum Dis 2008 67 1030 1033 18223265 10.1136/ard.2007.082123

[B32] Ozen S Ruperto N Dillon MJ Bagga A Barron K Davin JC Kawasaki T Lindsley C Petty RE Prieur AM EULAR/PReS endorsed consensus criteria for the classification of childhood vasculitides Ann Rheum Dis 2006 65 936 941 16322081 10.1136/ard.2005.046300 PMC1798210

[B33] Arnaud L Haroche J Toledano D Cacoub P Mathian A Costedoat-Chalumeau N Le Thi Huong-Boutin D Cluzel P Gorochov G Amoura Z Cluster analysis of arterial involvement in Takayasu arteritis reveals symmetric extension of the lesions in paired arterial beds Arthritis Rheum 2011 63 1136 1140 10.1002/art.30240 21452331

[B34] Yamada I Nakagawa T Himeno Y Kobayashi Y Numano F Shibuya H Takayasu arteritis: diagnosis with breath-hold contrast-enhanced three-dimensional MR angiography J Magn Reson Imaging 2000 11 481 487 10.1002/(SICI)1522-2586(200005)11:5<481::AID-JMRI3>3.0.CO;2-4 10813857

[B35] Garg SK Mohan S Kumar S Diagnostic value of 3D contrast-enhanced magnetic resonance angiography in Takayasu's arteritis--a comparative study with digital subtraction angiography Eur Radiol 2011 21 8 1658 1666 10.1007/s00330-011-2090-x 21344303

[B36] Andrews J Al-Nahhas A Pennell DJ Hossain MS Davies KA Haskard DO Mason JC Non-invasive imaging in the diagnosis and management of Takayasu's arteritis Ann Rheum Dis 2004 63 995 1000 10.1136/ard.2003.015701 15249328 PMC1755083

[B37] Li D Lin J Yan F Detecting disease extent and activity of Takayasu arteritis using whole-body magnetic resonance angiography and vessel wall imaging as a 1-stop solution J Comput Assist Tomogr 2011 35 468 474 10.1097/RCT.0b013e318222d698 21765303

[B38] Desai MY Stone JH Foo TK Hellmann DB Lima JA Bluemke DA Delayed contrast-enhanced MRI of the aortic wall in Takayasu's arteritis: initial experience AJR Am J Roentgenol 2005 184 1427 1431 15855090 10.2214/ajr.184.5.01841427

[B39] Choe YH Han BK Koh EM Kim DK Do YS Lee WR Takayasu's arteritis: assessment of disease activity with contrast-enhanced MR imaging AJR Am J Roentgenol 2000 175 505 511 10915704 10.2214/ajr.175.2.1750505

[B40] Jiang L Li D Yan F Dai X Li Y Ma L Evaluation of Takayasu arteritis activity by delayed contrast-enhanced magnetic resonance imaging Int J Cardiol 2012 155 2 262 267 10.1016/j.ijcard.2010.10.002 21059475

[B41] Tso E Flamm SD White RD Schvartzman PR Mascha E Hoffman GS Takayasu arteritis: utility and limitations of magnetic resonance imaging in diagnosis and treatment Arthritis Rheum 2002 46 1634 1642 10.1002/art.10251 12115196

[B42] Hoffman GS Takayasu arteritis: lessons from the American National Institutes of Health experience Int J Cardiol 1996 54 Suppl S99 102 9119532 10.1016/s0167-5273(96)88778-x

[B43] Liang KP Chowdhary VR Michet CJ Miller DV Sundt TM Connolly HM Crowson CS Matteson EL Warrington KJ Noninfectious ascending aortitis: a case series of 64 patients J Rheumatol 2009 36 2290 2297 10.3899/jrheum.090081 19648309

[B44] Khosroshahi A Stone JH A clinical overview of IgG4-related systemic disease Curr Opin Rheumatol 2011 23 57 66 10.1097/BOR.0b013e3283418057 21124086

[B45] Miller DV Maleszewski JJ The pathology of large-vessel vasculitides Clin Exp Rheumatol 2011 29 S92 98 21586202

[B46] Stone JR Aortitis, periaortitis, and retroperitoneal fibrosis, as manifestations of IgG4-related systemic disease Curr Opin Rheumatol 2010 23 88 94 21037477 10.1097/BOR.0b013e3283412f7c

[B47] Schmidt J Sunesen K Kornum JB Duhaut P Thomsen RW Predictors for pathologically confirmed aortitis after resection of the ascending aorta: a 12-year Danish nationwide population-based cross-sectional study Arthritis Res Ther 2011 13 R87 10.1186/ar3360 21676237 PMC3218902

[B48] Rojo-Leyva F Ratliff NB 3rd Cosgrove DM Hoffman GS Study of 52 patients with idiopathic aortitis from a cohort of 1,204 surgical cases Arthritis Rheum 2000 43 901 907 10.1002/1529-0131(200004)43:4<901::AID-ANR23>3.0.CO;2-U 10765937

[B49] Newburger JW Takahashi M Gerber MA Gewitz MH Tani LY Burns JC Shulman ST Bolger AF Ferrieri P Baltimore RS Diagnosis, treatment, and long-term management of Kawasaki disease: a statement for health professionals from the Committee on Rheumatic Fever, Endocarditis and Kawasaki Disease, Council on Cardiovascular Disease in the Young American Heart Association. Circulation 2004 110 2747 2771 10.1161/01.CIR.0000145143.19711.78 15505111

[B50] Kato H Sugimura T Akagi T Sato N Hashino K Maeno Y Kazue T Eto G Yamakawa R Long-term consequences of Kawasaki disease. A 10- to 21-year follow-up study of 594 patients Circulation 1996 94 1379 1385 10.1161/01.CIR.94.6.1379 8822996

[B51] Gordon JB Kahn AM Burns JC When children with Kawasaki disease grow up: myocardial and vascular complications in adulthood J Am Coll Cardiol 2009 54 1911 1920 10.1016/j.jacc.2009.04.102 19909870 PMC2870533

[B52] Nakamura Y Aso E Yashiro M Uehara R Watanabe M Oki I Yanagawa H Mortality among persons with a history of kawasaki disease in Japan: mortality among males with cardiac sequelae is significantly higher than that of the general population Circ J 2008 72 134 138 10.1253/circj.72.134 18159114

[B53] Margossian R Lu M Minich LL Bradley TJ Cohen MS Li JS Printz BF Shirali GS Sleeper LA Newburger JW Colan SD Predictors of coronary artery visualization in Kawasaki disease J Am Soc Echocardiogr 2011 24 53 59 10.1016/j.echo.2010.10.015 21172596 PMC3022385

[B54] Greil GF Seeger A Miller S Claussen CD Hofbeck M Botnar RM Sieverding L Coronary magnetic resonance angiography and vessel wall imaging in children with Kawasaki disease Pediatr Radiol 2007 37 666 673 10.1007/s00247-007-0498-x 17541574

[B55] Tacke CE Kuipers IM Groenink M Spijkerboer AM Kuijpers TW Cardiac magnetic resonance imaging for noninvasive assessment of cardiovascular disease during the follow-up of patients with Kawasaki disease Circ Cardiovasc Imaging 2011 4 712 720 10.1161/CIRCIMAGING.111.965996 21921132

[B56] Greil GF Stuber M Botnar RM Kissinger KV Geva T Newburger JW Manning WJ Powell AJ Coronary magnetic resonance angiography in adolescents and young adults with kawasaki disease Circulation 2002 105 908 911 10.1161/hc0802.105563 11864916

[B57] Mavrogeni S Papadopoulos G Douskou M Kaklis S Seimenis I Baras P Nikolaidou P Bakoula C Karanasios E Manginas A Cokkinos DV Magnetic resonance angiography is equivalent to X-ray coronary angiography for the evaluation of coronary arteries in Kawasaki disease J Am Coll Cardiol 2004 43 649 652 10.1016/j.jacc.2003.08.052 14975477

[B58] Suzuki A Takemura A Inaba R Sonobe T Tsuchiya K Korenaga T Magnetic resonance coronary angiography to evaluate coronary arterial lesions in patients with Kawasaki disease Cardiol Young 2006 16 563 571 10.1017/S1047951106001168 17116270

[B59] Arnold R Ley S Ley-Zaporozhan J Eichhorn J Schenk JP Ulmer H Kauczor HU Visualization of coronary arteries in patients after childhood Kawasaki syndrome: value of multidetector CT and MR imaging in comparison to conventional coronary catheterization Pediatr Radiol 2007 37 998 1006 10.1007/s00247-007-0566-2 17768616

[B60] Han BK Lindberg J Overman D Schwartz RS Grant K Lesser JR Safety and accuracy of dual-source coronary computed tomography angiography in the pediatric population J Cardiovasc Comput Tomogr 2012 6 252 259 10.1016/j.jcct.2012.01.004 22732198

[B61] Mavrogeni S Bratis K Karanasios E Georgakopoulos D Kaklis S Varlamis G Kolovou G Douskou M Papadopoulos G CMR evaluation of cardiac involvement during the convalescence of Kawasaki disease JACC Cardiovasc Imaging 2011 4 1140 1141 10.1016/j.jcmg.2011.04.021 21999875

[B62] Newburger JW Takahashi M Gerber MA Gewitz MH Tani LY Burns JC Shulman ST Bolger AF Ferrieri P Baltimore RS Diagnosis, treatment, and long-term management of Kawasaki disease: a statement for health professionals from the Committee on Rheumatic Fever, Endocarditis, and Kawasaki Disease, Council on Cardiovascular Disease in the Young American Heart Association. Pediatrics 2004 114 1708 1733 10.1542/peds.2004-2182 15574639

[B63] Henegar C Pagnoux C Puechal X Zucker JD Bar-Hen A Le Guern V Saba M Bagneres D Meyer O Guillevin L A paradigm of diagnostic criteria for polyarteritis nodosa: analysis of a series of 949 patients with vasculitides Arthritis Rheum 2008 58 1528 1538 10.1002/art.23470 18438816

[B64] Mavrogeni S Manoussakis MN Karagiorga TC Douskou M Panagiotakos D Bournia V Cokkinos DV Moutsopoulos HM Detection of coronary artery lesions and myocardial necrosis by magnetic resonance in systemic necrotizing vasculitides Arthritis Rheum 2009 61 1121 1129 10.1002/art.24695 19644909

[B65] Kobayashi H Yokoe I Hattan N Ohta H Nakajima Y Kobayashi Y Cardiac magnetic resonance imaging in polyarteritis nodosa J Rheumatol 2010 37 2427 2429 10.3899/jrheum.100450 21041263

[B66] Zöller B Li X Sundquist J Sundquist K Risk of subsequent coronary heart disease in patients hospitalized for immune-mediated diseases: a nationwide follow-up study from Sweden PLoS One 2012 7 e33442 10.1371/journal.pone.0033442 22438933 PMC3306397

[B67] Sarica-Kucukoglu R Akdag-Kose A Kayabal IM Yazganoglu KD Disci R Erzengin D Azizlerli G Vascular involvement in Behcet's disease: a retrospective analysis of 2319 cases Int J Dermatol 2006 45 919 921 10.1111/j.1365-4632.2006.02832.x 16911374

[B68] Calamia KT Schirmer M Melikoglu M Major vessel involvement in Behcet's disease: an update Curr Opin Rheumatol 2011 23 24 31 10.1097/BOR.0b013e3283410088 21124084

[B69] Geri G Wechsler B Thi Huong Du L Isnard R Piette JC Amoura Z Resche-Rigon M Cacoub P Saadoun D Spectrum of cardiac lesions in Behcet disease: a series of 52 patients and review of the literature Medicine (Baltimore) 2012 91 25 34 10.1097/MD.0b013e3182428f49 22198500

[B70] Szczeklik W Miszalski-Jamka T Mastalerz L Sokolowska B Dropinski J Banys R Hor KN Mazur W Musial J Multimodality assessment of cardiac involvement in Churg-Strauss syndrome patients in clinical remission Circ J 2011 75 649 655 10.1253/circj.CJ-10-0772 21139253

[B71] Vinit J Bielefeld P Muller G Pfitzenmeyer P Bonniaud P Lorcerie B Besancenot JF Heart involvement in Churg-Strauss syndrome: retrospective study in French Burgundy population in past 10 years Eur J Intern Med 2010 21 341 346 10.1016/j.ejim.2010.05.004 20603049

[B72] Wassmuth R Gobel U Natusch A Schneider W Kettritz R Dietz R Luft FC Schulz-Menger J Cardiovascular magnetic resonance imaging detects cardiac involvement in Churg-Strauss syndrome J Card Fail 2008 14 856 860 10.1016/j.cardfail.2008.07.227 19041050

[B73] Chun W Grist TM Kamp TJ Warner TF Christian TF Images in cardiovascular medicine, Infiltrative eosinophilic myocarditis diagnosed and localized by cardiac magnetic resonance imaging Circulation 2004 110 e19 10.1161/01.CIR.0000135586.94417.3C 15262855

[B74] Petersen SE Kardos A Neubauer S Subendocardial and papillary muscle involvement in a patient with Churg-Strauss syndrome, detected by contrast enhanced cardiovascular magnetic resonance Heart 2005 91 e9 10.1136/hrt.2004.050070 15604316 PMC1768616

[B75] Su HS Nahrendorf M Panizzi PR Breckwoldt MO Rodriguez E Iwamoto Y Aikawa E Weissleder R Chen JW Vasculitis: molecular imaging by targeting the inflammatory enzyme myeloperoxidase Radiology 2011 262 1 181 90 22084204 10.1148/radiol.11110040 PMC3244672

